# Adherence of Free-Tier Large Language Models to the 2024 European Society of Cardiology (ESC) Guidelines for the Management of Elevated Blood Pressure and Hypertension: A Comparative Study

**DOI:** 10.7759/cureus.104111

**Published:** 2026-02-23

**Authors:** Aleksander Polus, Dawid Boczkowski, Rania Suleiman, Bartosz Palacz, Natalia Marianna Kubis, Julia Anna Wrona, Wiktor Perz, Maria Magdalena Teper, Anhelina Korolchuk, Jedrzej Piotrowski, Anna Gluzicka, Anna Matyas, Aleksander Tuteja, Piotr Sawina, Aleksandra Wielochowska

**Affiliations:** 1 Internal Medicine, Medical University of Lodz, Lodz, POL; 2 Internal Medicine, Central Teaching Hospital, Medical University of Lodz, Lodz, POL; 3 Medicine, Wrocław Medical University, Wroclaw, POL; 4 College of Medicine, Jan Kochanowski University of Kielce, Kielce, POL; 5 Medicine, Independent Public Health Care Institution, Ministry of Interior Affairs and Administration, Kielce, POL; 6 Medicine, Jan Kochanowski University of Kielce, Kielce, POL; 7 Medicine, Independent Public Health Care Institution, Ministry of Interior Affairs and Administration, Krakow, POL; 8 Medicine, Copernicus Memorial Hospital, Lodz, POL; 9 Medicine, Voivodeship Combined Hospital, Kielce, POL; 10 Medicine, Medical University of Lodz, Lodz, POL; 11 Medicine, Medical University of Silesia, Katowice, POL; 12 Internal Medicine, Non-Public Health Care Institution (NZOZ) Hospital, Dzierzoniow, POL; 13 Medicine, Central Teaching Hospital, Medical University of Lodz, Lodz, POL

**Keywords:** cardiology research, elevated blood pressure, esc guidelines, hypertension awareness, large language models, llms

## Abstract

Background

Hypertension remains the leading modifiable risk factor for cardiovascular disease and premature death worldwide. In 2024, the European Society of Cardiology (ESC) released updated guidelines for the management of elevated blood pressure and hypertension. Concurrently, the integration of artificial intelligence into healthcare has accelerated, with large language models (LLMs) becoming accessible tools for information retrieval.

Objective

This study aims to evaluate and compare the accuracy and adherence of three popular free-tier LLMs (ChatGPT-5.2, Gemini 3 Flash, and Claude 4.5 Sonnet) in responding to questions based strictly on the 2024 ESC Guidelines.

Methods

We conducted a comparative cross-sectional study in January 2026 to evaluate the performance of three LLMs. The primary source of ground truth was the 2024 ESC Guidelines. A dataset of 40 specific questions was generated, covering key domains including diagnosis, treatment targets, lifestyle modifications, and comorbidities. Questions comprised both factual queries and clinical case reports. Responses were categorized by a qualified physician as correct, inaccurate, or incorrect based strictly on guidelines. Statistical analysis was performed using the Fisher-Freeman-Halton exact test to evaluate differences in performance.

Results

The overall accuracy across all models was high, with no statistically significant differences in performance observed (p>0.99). Claude 4.5 Sonnet achieved the highest numerical accuracy, providing correct responses to 33 out of 40 questions (82.5%). ChatGPT-5.2 and Gemini 3 Flash achieved identical correctness rates of 80.0% (32 out of 40 correct answers). A qualitative analysis revealed a distinct tendency toward overly aggressive management in complex clinical scenarios, suggesting a "safety bias" where models default to intensive intervention rather than nuanced guideline steps.

Conclusions

The evaluated free-tier LLMs demonstrated comparable and high proficiency in interpreting the 2024 ESC Guidelines. Despite this potential, the study identified a recurrent safety bias manifesting as a tendency toward over-medicalization. While these models serve as promising auxiliary tools for medical education, verification of AI-generated advice against official guideline documents remains essential.

## Introduction

Hypertension remains the leading modifiable risk factor for cardiovascular disease and premature death worldwide, affecting over one billion adults [1]. The effective management of blood pressure is critical for reducing the burden of stroke, myocardial infarction, and heart failure [2]. In 2024, the European Society of Cardiology (ESC) released updated guidelines for the management of elevated blood pressure and hypertension, introducing significant changes in blood pressure categorization, treatment targets, and implementation strategies compared to previous iterations [3].

Concurrently, the integration of artificial intelligence (AI) into healthcare has accelerated. Large Language Models (LLMs) have become increasingly accessible to both medical professionals and patients as tools for information retrieval, clinical decision support, and even as assistants to academic teachers [4,5]. Recent studies have demonstrated the capability of LLMs to pass medical licensing examinations and provide accurate responses to clinical queries [6]. However, concerns regarding "hallucinations," which are plausible-sounding but incorrect information, persist, particularly when models interpret complex or recently updated clinical guidelines [7,8].

One of the most popular LLMs is ChatGPT (made by OpenAI, San Francisco, California, United States). Optimized for dialogue, this model employs a method known as Reinforcement Learning from Human Feedback (RLHF) to align the system's responses with human instructions. As a sibling model to InstructGPT, the conversational format enables ChatGPT to answer follow-up questions, admit its mistakes, challenge incorrect premises, and reject inappropriate requests [9].

This study also evaluated Gemini 3 Flash (Google, Mountain View, California, United States), a model released in late 2025. This system employs a natively multimodal architecture, enabling it to process and generate content across various formats, including text and images. The model is optimized for high-volume, low-latency tasks, designed to balance computational efficiency with the reasoning capabilities required for analyzing complex inputs [10].

Finally, the study examined Claude 4.5 Sonnet (Anthropic, San Francisco, California, United States). This model utilizes a training methodology focused on safety and alignment, often referred to as "Constitutional AI," to reduce the likelihood of generating erroneous outputs. The system is designed to balance processing speed with reasoning depth, aiming to provide solid analysis of complex inputs while maintaining computational efficiency [11].

Therefore, the aim of this study is to evaluate and compare the accuracy and adherence of three popular free-tier LLMs (ChatGPT-5.2, Gemini 3 Flash, and Claude 4.5 Sonnet) in responding to questions based strictly on the 2024 ESC Guidelines.

## Materials and methods

We conducted a comparative cross-sectional study in January 2026 to evaluate the performance of three LLMs. The primary source of ground truth was the 2024 ESC Guidelines for the Management of Elevated Blood Pressure and Hypertension [3]. A dataset of 40 specific questions was generated by the authors, covering key domains of the guidelines, including diagnosis, treatment targets, lifestyle modifications, and specific comorbidities (e.g., pregnancy and chronic kidney disease). The questions comprised both factual queries and clinical case reports to simulate real-world medical decision-making.

The selection of LLMs for this study was guided by two primary inclusion criteria: (1) the availability of a functional "free-tier" version accessible to the general public without a paid subscription and (2) the capability to access real-time information via the internet to ensure the retrieval of the most current medical data. Based on these criteria, three state-of-the-art models were included in the final analysis: ChatGPT-5.2 (OpenAI), Gemini 3 Flash (Google), and Claude 4.5 Sonnet (Anthropic).

To ensure methodological consistency and mitigate the risk of context retention bias, each query was executed in a newly initialized chat session. A standardized prompt engineering strategy was employed; every question was prefaced with the specific instruction: "You are an expert cardiologist. Based STRICTLY on the 2024 ESC Guidelines for the Management of Elevated Blood Pressure and Hypertension..." Furthermore, the questions were rigorously formulated to elicit objective facts derived exclusively from the 2024 ESC Guidelines for the Management of Elevated Blood Pressure and Hypertension, thereby precluding the need for subjective clinical opinion or external medical knowledge. All generated responses were systematically recorded in a structured database and subsequently evaluated by a qualified physician.

Subsequently, the physician categorized each response into one of three mutually exclusive groups based on its adherence to the source text: (1) Correct: responses that were comprehensive and fully consistent with the 2024 ESC Guidelines; (2) Inaccurate: responses that contained truthful elements but were incomplete, lacked necessary detail, or failed to directly address the specific query; and (3) Incorrect: responses that explicitly contradicted the recommendations provided in the 2024 ESC Guidelines.

Statistical analysis was performed using Jamovi software (Version 2.7), based on the R statistical language [12]. Categorical variables, representing response accuracy (categorized as correct, inaccurate, or incorrect), were summarized as absolute frequencies (n) and percentages (%). To evaluate differences in performance between the three language models, the distribution of responses was compared using the Fisher-Freeman-Halton exact test (an extension of Fisher's exact test for contingency tables larger than 2 x 2). This method was selected due to the limited sample size (N=40 per group) and low expected cell counts in the error categories. A two-tailed p-value of less than 0.05 was considered statistically significant. The analysis revealed no statistically significant differences in accuracy rates between the evaluated models (p>0.99).

## Results

The study evaluated a total of 120 responses (40 per model) generated by three LLMs: ChatGPT-5.2, Gemini 3 Flash, and Claude 4.5 Sonnet. The overall accuracy across all models was high, with no statistically significant differences in performance observed (p>0.99) (Table 1).

**Table 1:** Evaluation of response accuracy for ChatGPT-5.2, Gemini 3 Flash, and Claude 4.5 Sonnet. Fisher's exact test (p>0.99) indicates comparable performance across all three models.

Model	Correct	Inaccurate	Incorrect	Total (n)
GPT-5.2	32	4	4	40
Gemini 3 Flash	32	5	3	40
Claude Sonnet 4.5	33	4	3	40
Total	97	13	10	120
Fisher's Exact Test	p>0.99			

**Figure 1:**
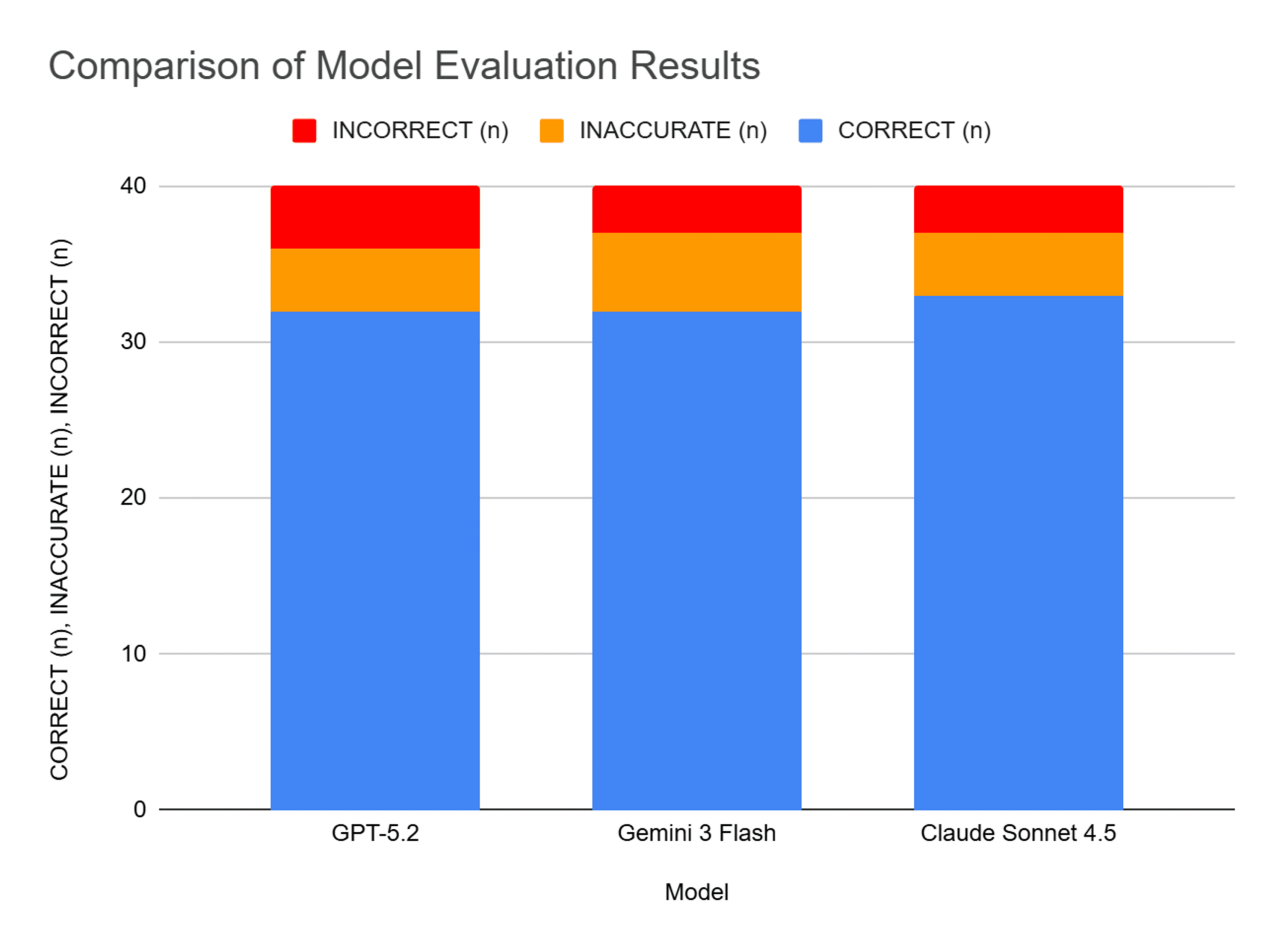
Comparison of model evaluation results.

Claude 4.5 Sonnet achieved the highest numerical accuracy, providing correct responses to 33 out of 40 questions (82.5%). It generated four inaccurate responses (10.0%) and three incorrect responses (7.5%). ChatGPT-5.2 and Gemini 3 Flash achieved identical correctness rates of 80.0% (32 correct answers). However, the distribution of errors varied slightly; ChatGPT-5.2 produced four inaccurate (10.0%) and four incorrect (10.0%) responses, whereas Gemini 3 Flash yielded five inaccurate (12.5%) and three incorrect (7.5%) answers (Table 2, Figure 1).

**Table 2:** Comparison of correct, inaccurate, and incorrect response rates for ChatGPT-5.2, Gemini 3 Flash, and Claude 4.5 Sonnet, showing a slight performance lead for Claude 4.5 Sonnet.

Result category	GPT-5.2 total	Gemini 3 Flash total	Claude Sonnet 4.5 total
Correct (n)	32	32	33
Inaccurate (n)	4	5	4
Incorrect (n)	4	3	3
Inaccuracy rate (%)	10%	12.5%	10%
Correctness rate (%)	80%	80%	82.5%
Incorrect rate (%)	10%	7.5%	7.5%

Regarding the specific domains of the 2024 ESC Guidelines, all models demonstrated robust performance in answering factual questions about treatment targets and definitions. The majority of errors (classified as inaccurate or incorrect) occurred in complex clinical scenarios requiring multistep reasoning or specific cutoff values for risk stratification. No specific pattern of failure unique to one model was identified; errors were generally distributed across different guideline sections.

## Discussion

This study demonstrates that modern, free-tier LLMs have achieved a high level of proficiency in interpreting the 2024 ESC Guidelines for the Management of Elevated Blood Pressure and Hypertension. With accuracy rates ranging from 80% to 82.5%, these tools show potential as supplementary resources for medical education and information retrieval.

Our findings align with the growing body of literature, particularly the exploratory study by Sarraju et al., which evaluated AI model responses to cardiovascular disease prevention questions [13]. They reported an appropriateness rate of 84% for simple queries, a figure comparable to the accuracy rates of 80-82.5% observed in our analysis.

Similarly, Kung et al. evaluated ChatGPT on the United States Medical Licensing Examination (USMLE), observing moderate accuracy rates that approached passing performance, ranging from approximately 36% to 75% depending on the exam step and question format. Although these figures are lower than the >80% adherence observed in our study, the authors emphasized that the model displayed high internal concordance (94.6%) and produced at least one significant insight in 88.9% of its explanations. This high "density of insight" supports the conclusion that LLMs have the potential to augment human learning in medical education, even if their standalone diagnostic accuracy is not yet perfect [14].

A qualitative analysis of the erroneous responses reveals a distinct tendency toward overly aggressive management, often bypassing nuanced guideline steps in favor of immediate intervention. This pattern was evident in question six and question 10, where models frequently suggested immediate pharmacological treatment for a patient with elevated blood pressure and high cardiovascular risk. In contrast, the 2024 ESC Guidelines explicitly prioritize a three-month trial of lifestyle interventions before initiating drug therapy in this specific clinical scenario. Similarly, in question seven regarding severe hypertension in pregnancy, the models interpreted the situation as requiring mandatory, urgent hospitalization. However, the guidelines classify hospitalization in this context as a class IIa recommendation ("should be considered") rather than an absolute imperative. This behavior suggests a "safety bias" inherent in the models, where the AI defaults to the most intensive course of action to minimize perceived risk, which could potentially lead to over-medicalization and unnecessary strain on healthcare resources (Table 3).

**Table 3:** Selected questions that exemplify overly aggressive therapy or treatment.

Question ID	Question content	Response: GPT-5.2	Response: Gemini 3 Flash	Response: Claude Sonnet 4.5
Q6	"You are an expert cardiologist. Based STRICTLY on the 2024 ESC Guidelines, advise on the management. Patient: 62-year-old male. Comorbidities: History of Myocardial Infarction (CAD). BP: Consistently 135/85 mmHg. Question: According to the 2024 Guidelines, is pharmacological blood pressure lowering treatment recommended for this specific patient? (Yes/No)"	Answer (based strictly on the 2024 ESC Guidelines): Yes. According to the 2024 European Society of Cardiology (ESC) Guidelines for the management of elevated blood pressure and hypertension, patients with established cardiovascular disease (including coronary artery disease and prior myocardial infarction) are recommended to receive pharmacological blood pressure-lowering treatment when their confirmed blood pressure is ≥130/80 mmHg to reduce future cardiovascular risk. This applies even if office BP is below the traditional hypertension cutoff of ≥140/90 mmHg.	Yes. The 2024 guidelines introduced a new classification and more intensive treatment triggers for patients with high cardiovascular risk. With a BP of 135/85 mmHg, the patient falls into the "Elevated BP" category (defined as 120–139/70–89 mmHg). The patient has established Coronary Artery Disease (CAD) due to a history of Myocardial Infarction. This automatically places him in the "Increased Cardiovascular Risk" category. For individuals with "Elevated BP" who are at increased cardiovascular risk (including those with established CVD like CAD), pharmacological treatment is now recommended if their BP is equal or above 130/80 mmHg after lifestyle advice (or alongside it).	Answer: YES According to the 2024 ESC Guidelines, pharmacological blood pressure lowering treatment IS recommended for this patient. Here's why: Patient's BP Classification: The patient has BP of 135/85 mmHg, which falls into the "Elevated BP" category (defined as office SBP 120-139 mmHg or DBP 70-89 mmHg in the 2024 guidelines). High-Risk Condition Present: The patient has a history of myocardial infarction (coronary artery disease), which is classified as established cardiovascular disease and represents a "sufficiently high CVD risk condition" Treatment Recommendation: The 2024 ESC guidelines recommend pharmacological therapy for individuals with elevated BP (SBP 130-139 or DBP 80-89 mmHg) who have high-risk CVD conditions including established CVD (Class I recommendation Specific to CAD: The guidelines specifically mention that individuals with history of CVD, predominantly coronary artery disease, should receive pharmacological BP-lowering treatment even when BP is in the elevated range
Q7	"You are an expert cardiologist. Based STRICTLY on the ESC 2024 Guidelines, answer the question. A pregnant woman comes to her gynecologist for a checkup. Her physical examination reveals an abnormal BP of 166/121 mmHg. A repeat measurement reveals a BP of 162/100 mmHg. She reports no other symptoms. What should be the next course of action, according to the 2024 ESC Guidelines for the Management of Elevated Blood Pressure and Hypertension?"	This pregnant woman has severe hypertension (BP ≥160/110 mmHg). Immediate action is required. Next course of action: Treat as a hypertensive emergency in pregnancy, Urgent hospital admission, Immediate evaluation for pre-eclampsia (labs, proteinuria, maternal and fetal assessment), Prompt initiation of antihypertensive therapy with pregnancy-safe agents (e.g. labetalol or nifedipine), Close maternal and fetal monitoring	As an expert cardiologist following the 2024 ESC Guidelines for the Management of Elevated Blood Pressure and Hypertension, the case of this patient requires immediate and specific action. Based on the measurements provided (166/121 mmHg and 162/100 mmHg), the patient is presenting with confirmed severe hypertension (defined in pregnancy as SBP above or equal 160 mmHg and/or DBP 110 mmHg). You should refer the patient for urgent hospital admission for the initiation of antihypertensive therapy (typically intravenous labetalol or oral nifedipine/methyldopa depending on the clinical urgency) and a full pre-eclampsia workup.	This pregnant woman presents with severe acute hypertension in pregnancy (BP >160/110 mmHg on repeated measurements). According to the guidelines, acute onset of severe hypertension exceeding 160/110 mmHg persisting for more than 15 minutes is considered an emergency Practice Nurse, even in the absence of other symptoms. The gynecologist should immediately refer this patient for hospital admission and initiate acute BP-lowering treatment. This is classified as a hypertensive emergency in pregnancy requiring prompt intervention to prevent maternal stroke and adverse perinatal outcomes. First-line antihypertensive agents in pregnancy include labetalol, alpha-methyldopa, or extended-release nifedipine
Q10	"You are an expert cardiologist. Based STRICTLY on the 2024 ESC Guidelines, advise on the management. A 61-year-old woman presented to her physician for a routine checkup. Her BP was 133/69 mmHg on physical examination. She had a history of type 2 diabetes treated with metformin and obesity. Does the patient require antihypertensive medication, as per the 2024 ESC guidelines for the management of elevated blood pressure and hypertension?"	Yes — according to the 2024 ESC guidelines, this 61-year-old woman with diabetes and elevated BP (133/69 mmHg) is a candidate for antihypertensive medication if elevated BP is confirmed and persists ≥130/80 mmHg after lifestyle intervention due to her high cardiovascular risk profile. However, the first step is confirmation and lifestyle optimization before starting drugs.	Yes, the patient is a candidate for antihypertensive medication. While her BP (133/69 mmHg) is not "hypertensive" by old standards (>140/90), it is now classified as Elevated BP. Because she has Type 2 Diabetes, she is automatically considered high cardiovascular risk, which warrants pharmacological treatment to prevent future heart disease or stroke.	Antihypertensive medication SHOULD be initiated along with lifestyle modifications (weight reduction, dietary sodium restriction, physical activity).

This inclination toward risk aversion is not unique to the present study but aligns with broader findings in recent literature regarding AI performance in clinical settings. For instance, Levine et al. (2023) demonstrated that while LLMs can achieve high diagnostic accuracy, they exhibit significant “over-triage” in management decisions, frequently recommending emergency care for conditions that could be safely managed in primary care settings [15]. Similarly, Hirosawa et al. (2023) observed a comparable trend in diagnostic reasoning, noting that AI models displayed a propensity for ordering excessive and unnecessary diagnostic tests. This phenomenon, often driven by the safety alignment training of the models, corroborates the hypothesis that current AI iterations default to a defensive medical strategy, prioritizing theoretical safety over guideline-adherent resource stewardship [16].

Finally, the accessibility of these tools cannot be overlooked. We specifically evaluated free-tier models to reflect real-world access for the general public. While paid versions might offer superior reasoning, the widespread availability of free versions necessitates understanding their limitations. As noted by Meskó and Topol, the democratization of AI in healthcare brings both opportunities for patient empowerment and risks of misinformation, underscoring the need for digital health literacy [17].

## Conclusions

This study has several limitations. First, the dataset of 40 questions, while covering key guideline domains, is relatively small, which may limit the statistical power to detect minor differences between models. Second, the evaluation was conducted at a specific point in time (January 2026); given the rapid update cycles of LLMs, performance may change within weeks. Additionally, LLMs are nondeterministic; rerunning the prompts might yield slightly different phrasing.

In conclusion, the evaluated free-tier LLMs (ChatGPT-5.2, Gemini 3 Flash, and Claude 4.5 Sonnet) demonstrated comparable and high proficiency in interpreting the 2024 ESC Guidelines, achieving accuracy rates between 80% and 82.5%. Despite this potential, the study identified a recurrent "safety bias" manifesting as a tendency toward over-medicalization and aggressive treatment recommendations that deviate from the nuanced, stepwise approach of the guidelines. Therefore, while these models serve as promising auxiliary tools for medical education and rapid information retrieval, they currently lack the precision required for autonomous clinical practice. Verification of AI-generated advice against official guideline documents remains essential for safe patient care.
